# Dual Regulatory Roles of SlGAMYB1 in Tomato Development: GA-Dependent and GA-Independent Mechanisms

**DOI:** 10.3390/plants14111613

**Published:** 2025-05-25

**Authors:** Fanjia Zhong, Fengpan Wang, Zike Chen, Tengbo Huang, Panpan Zhao

**Affiliations:** 1 Guangdong Provincial Key Laboratory for Plant Epigenetics, College of Life Sciences and Oceanography, Shenzhen University, Shenzhen 518060, China; zhongfanjia2021@email.szu.edu.cn (F.Z.); kekedadada2021@163.com (Z.C.); tengbohuang@szu.edu.cn (T.H.); 2 The Orchid Conservation and Research Center of Shenzhen, Shenzhen 518060, China; duibian@126.com

**Keywords:** floral organ formation, gibberellin, leaf development, *SlGAMYB1*, tomato

## Abstract

The R2R3-MYB transcription factor GAMYB plays crucial roles in plant growth and development, but the biological functions of *SlGAMYB1* in tomato remain poorly understood. Here, we investigated the roles of *SlGAMYB1* by overexpressing a miR159-resistant version (*35S:SlGAMYB1^m^*) in tomato. Transgenic plants exhibited a dwarf phenotype with reduced internode elongation, which was associated with decreased bioactive gibberellin (GA) levels due to transcriptional repression of *SlGA3ox1* and activation of *SlGA2ox1/2/4/5*. Additionally, *35S:SlGAMYB1^m^* altered leaf morphology by inhibiting cell proliferation through downregulation of cell cycle genes, resulting in larger but fewer epidermal cells. Intriguingly, *35S:SlGAMYB1^m^* plants displayed increased floral organ number, a process likely mediated by the upregulation of *SlWUS* rather than GA signaling. These findings demonstrate that *SlGAMYB1* regulates diverse aspects of tomato development through both GA-dependent and independent pathways, providing new insights into the functional diversification of *GAMYB* genes and potential strategies for genetic improvement of tomato architecture and yield.

## 1. Introduction

Plant growth and development is a complex process that is finely regulated by multiple endogenous hormones and transcription factors. Among these regulators, gibberellin (GA) stands out as a crucial plant hormone that plays a key role in various stages of plant growth [1,2]. GA is indispensable in promoting cell elongation, cell division, and the transition from vegetative to reproductive growth. For instance, GA significantly influences plant height, leaf morphology, flowering time, and floral organ development [3,4,5]. The biosynthesis and signaling pathways of GA have been extensively studied in many plant species, and it is well established that GA regulates plant growth and development by modulating the expression of a series of downstream genes [3].

Transcription factors are pivotal in the modulation of target gene expression, thereby orchestrating plant growth and development. The MYB family is one of the largest transcription factor families in plants [6] and can be divided into four major subclades—1R-MYB, R2R3-MYB, 3R-MYB, and 4R-MYB [7]. Among these, the R2R3-MYB subfamily, with its numerous members, plays a pivotal role in a variety of developmental and physiological processes, including tissue differentiation, metabolic pathways, and stress tolerance [8,9,10]. The GAMYB transcription factors belong to the R2R3-MYB family, serving as a positive regulator in the GA signaling pathway [11]. They are characterized by highly conserved R2R3 DNA-binding domains in their N-terminal regions, as well as distinctive motifs known as Box 1, Box 2, and Box 3, which are crucial for their function [12,13].

GAMYB transcription factors exhibit remarkable functional plasticity across plant species, orchestrating diverse developmental programs through GA-dependent and independent pathways. As pivotal components of GA signaling, these regulators were first characterized in barley (*Hordeum vulgare* L.) aleurone cells, where they mediate α-amylase activation during seed germination [14]. Subsequent studies have established their essential role in reproductive development, particularly during male organ formation, where GAMYB-mediated regulation of tapetal programmed cell death is crucial, a process whose disruption causes male sterility in key crop species such as rice (*Oryza sativa* L.), wheat (*Triticum aestivum* L.), and cucumber (*Cucumis sativus* L.) [11,12,15]. Beyond gametogenesis, GAMYB proteins demonstrate broad developmental influence, coordinating seed maturation through storage protein activation (e.g., *Hor2* and *Amy6.4* in barley), modulating architectural traits (including internode elongation and tiller formation in rice), and mediating stress responses (particularly drought adaptation in barley spike development) [16,17,18]. The regulatory repertoire of *GAMYB* genes extends to flowering time control, though with intriguing species-specific variations. In tomato (*Solanum lycopersicum* L.), the GAMYB homolog SlMYB33 appears to regulate floral transition through putative interactions with flowering-associated genes [4], while heterologous expression studies in Arabidopsis reveal that different wheat TaGAMYB isoforms can exert diametrically opposed effects on flowering timing [19]. This functional divergence highlights the evolutionary adaptability of GAMYB proteins as developmental regulators. Their capacity to integrate hormonal signals with environmental cues while maintaining tissue-specific functions positions GAMYB transcription factors as central nodes in the regulatory networks governing plant architecture, reproductive success, and stress-adaptation characteristics that make them particularly valuable targets for precision breeding strategies in crop improvement programs.

GAMYB transcription factors are well-established targets of microRNA159 (miR159), with their interaction playing a pivotal role in plant organ development [12]. Multiple GAMYB family members, including *MYB33*, *MYB65*, *MYB81*, *MYB97*, *MYB101*, *MYB104*, and *MYB120*, contain conserved miR159 binding sites and regulate diverse biological processes [12,20]. Among these, *MYB33* and *MYB65* serve as primary miR159 targets in Arabidopsis, where their double knockout results in severe developmental defects, including shortened filaments, pollen abortion, and male sterility [12,21]. This regulatory module is conserved across species: in strawberry (*Fragaria × ananassa*), Fa-miR159a/b coordinates with gibberellins to promote flower receptacle development [22], while in maize (*Zea mays* L.), zma-miR159-mediated regulation of *ZmMYB74* and *ZmMYB138* controls endosperm cell proliferation, ultimately affecting grain size and weight [23]. Similarly, in tomato, both *SlGAMYB1* and *SlGAMYB2* are validated targets of Sly-miR159 [24,25]. While *SlGAMYB2* has been extensively characterized as a regulator of fruit morphology through GA biosynthesis, flowering time, and pollen development, the functional significance of *SlGAMYB1* remains largely unexplored despite its similar targeting by Sly-miR159 [4,24]. Notably, Sly-miR159 overexpression studies that simultaneously downregulate both *SlGAMYB1* and *SlGAMYB2* result in precocious fruit initiation and parthenocarpy, suggesting potential overlapping yet distinct roles for these paralogs in tomato development [25]. This knowledge gap regarding *SlGAMYB1*’s specific functions, particularly in comparison to its well-studied counterpart *SlGAMYB2*, highlights the need for focused investigation to fully understand the GAMYB regulatory network in tomato.

Given the crucial roles of GA and GAMYBs in plant growth, we hypothesized that SlGAMYB1 influences organ development via GA signaling. Using a miR159-resistant SlGAMYB1 (*35S:SlGAMYB1^m^*), we found that it regulates plant height by suppressing *SlGA3ox1* and activating *SlGA2ox1/2/4/5*, reducing GA levels and causing dwarfism. It also alters leaf morphology by inhibiting cell proliferation and increases floral organ number independently of GA, likely via *SlWUS* upregulation. These findings reveal SlGAMYB1’s pleiotropic roles and potential breeding applications.

## 2. Results

### 2.1. Characterization and Expression Pattern of SlGAMYB1

**Figure 1:**
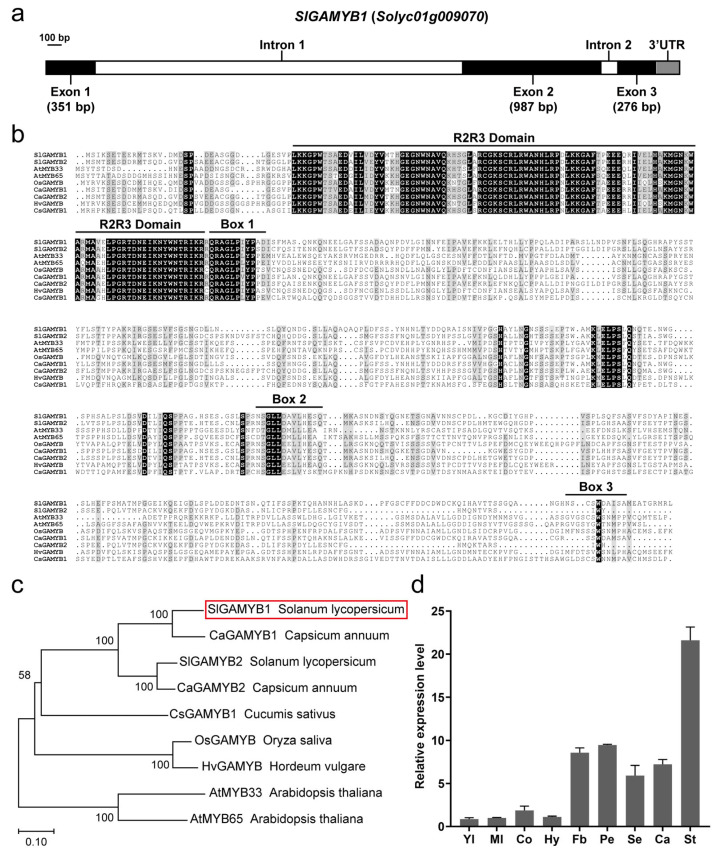
Structural, phylogenetic, and expression analyses of SlGAMYB1. (**a**) Gene structure of *SlGAMYB1*. The exons, 3’UTR, and introns are symbolized by dark boxes, a gray box, and white boxes, respectively. The scale bar represents 100 bp. (**b**) Multiple sequence alignment of GAMYB proteins. Alignment of SlGAMYB1 with other GAMYB proteins from tomato (SlGAMYB2), Arabidopsis (AtMYB33 and AtMYB65), rice (OsGAMYB), pepper (CaGAMYB1 and CaGAMYB2), barley (HvGAMYB), and cucumber (CsGAMYB1), highlighting the conserved R2R3 DNA-binding domains, Box 1, Box 2, and Box 3. The black background indicates identical amino acids among the aligned sequences. (**c**) Phylogenetic analysis of GAMYB homologs. Neighbor-joining tree showing the evolutionary relationships among GAMYB proteins from different species. SlGAMYB1 is highlighted by a red box, and the tree is scaled to a branch length of 0.1 substitutions per site. Bootstrap values (1000 replications) are indicated at the nodes. (**d**) Transcriptional patterns of the *SlGAMYB1* in tomato. The relative expression levels were measured by quantitative real-time PCR (qRT-PCR) and normalized to the expression of *ACTIN*. Error bars represent SEM (n = 3 in (**d**)). Tissues include young leaf (Yl), mature leaf (Ml), cotyledon (Co), hypocotyl (Hy), flower bud (Fb), petal (Pe), sepal (Se), carpel (Ca), and stamen (St).

Our previous study identified *SlGAMYB1* (*Solyc01g009070*) and *SlGAMYB2* (*Solyc06g073640*) as members of the R2R3MYB family, which are the major targets of miR159 [24,26]. Given the extensive documentation on the role of *SlGAMYB2*, our present investigation has delved into the relatively unexplored terrain of *SlGAMYB1* to unravel the specific contributions of *SlGAMYB1* to plant biology, complementing the existing body of knowledge with fresh insights into its distinct functions and regulatory mechanisms. The genomic fragment of *SlGAMYB1* consists of three exons and two introns, which encompass an open reading frame (ORF) of 1614 bp, encoding 537 amino acid residues. The lengths of the exons are 351 bp, 987 bp, and 276 bp. The gene structure is represented with exons in black, introns in white, and the 3’ UTR in gray (Figure 1a).

Sequence alignment of SlGAMYB1 with other GAMYB proteins reveals high conservation within the R2R3 DNA-binding domains, Box 1, Box 2, and Box 3 motifs (Figure 1b). The R2R3 DNA-binding domain displays an extraordinary degree of sequence conservation, with over 80% homology to its analogous segments in HvGAMYB from barley, OsGAMYB from rice, AtGAMYB from Arabidopsis thaliana, and CsGAMYB from cucumber.

To elucidate the evolutionary relationships among GAMYB proteins across various species, an unrooted neighbor-joining (NJ) phylogenetic tree was constructed according to the full-length protein sequences of six distinct species (Appendix A Table A3). As shown in Figure 1c, the GAMYB proteins neatly segregate into two major clusters. The distinct branching of OsGAMYB from rice and HvGAMYB from barley implies a potential functional diversification of GAMYB genes within different species. Notably, the GAMYB proteins from Solanaceae family members, encompassing tomato and pepper (*Capsicum annuum* L.), coalesce into a singular group. This aggregation hints at a shared ancestral lineage and potentially overlapping biological functions. Among these sequences, the tomato GAMYBs (SlGAMYB1 and SlGAMYB2), pepper GAMYBs (CaGAMYB1 and CaGAMYB2), and the cucumber GAMYB (CsGAMYB1) are nestled within the same clade. This clade stands in stark contrast to the clade occupied by the Arabidopsis thaliana MYB33 and MYB65 proteins, underscoring significant evolutionary divergence.

**Figure A1:**
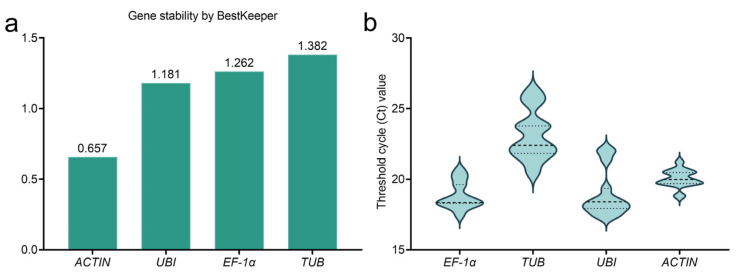
Stability analysis of reference genes. (**a**) Stability assessment of candidate reference genes using BestKeeper. Lower values indicate higher stability, with genes ranked from most stable (*ACTIN*) to least stable (*TUB*). (**b**) Threshold cycle (Ct) values of candidate reference genes. Lower Ct values correlate with higher expression levels, ordered from highest (*EF-1a*) to lowest (*ACTIN*). Data represent mean values from technical replicates. The thick dashed line represents the median (50th percentile), indicating the central tendency of the data. The thin dashed lines denote the first (25th percentile) and third (75th percentile) quartiles, reflecting the variability and spread of the data.

To explore the potential role of *SlGAMYB1* in tomato development, we conducted a comprehensive analysis of its expression across various vegetative and reproductive tissues using quantitative real-time reverse transcription polymerase chain reaction (qRT-PCR). Prior to target gene quantification, we rigorously evaluated four candidate reference genes for normalization accuracy, including *ACTIN*, *Ubiquitin* (*UBI*), *Elongation factor-1 alpha* (*EF-1α*), and *Tubulin* (*TUB*). Ct values from all tissues (Figure 1d and Figure A1b) were analyzed using RefFinder software [27], which integrates multiple algorithms to assess gene stability. The composite stability ranking (Figure A1a) identified *ACTIN* as the most stable reference (lowest score), followed by *UBI*, *EF-1α*, and *TUB*, establishing *ACTIN* as the optimal internal control for subsequent *SlGAMYB1* expression profiling across diverse tissue types.

Our results yielded insights into the tissue-specific expression dynamics of *SlGAMYB1*, indicating a ubiquitous presence across all sampled tissues, albeit with a discernible heterogeneity in expression intensities. Notably, *SlGAMYB1* transcripts were particularly pronounced in reproductive tissues, including flower buds, sepals, petals, stamens, and carpels, while it was less abundant in vegetative tissues such as young and mature leaves, cotyledons, and hypocotyls (Figure 1d). These findings suggest that SlGAMYB1 may serve as a regulatory hub in the complex interplay of developmental processes, with a potentially significant impact on the reproductive phase in tomato.

### 2.2. Overexpression of SlGAMYB1 Results in Plant Dwarfism via GA Deficiency in Tomato

**Figure 2:**
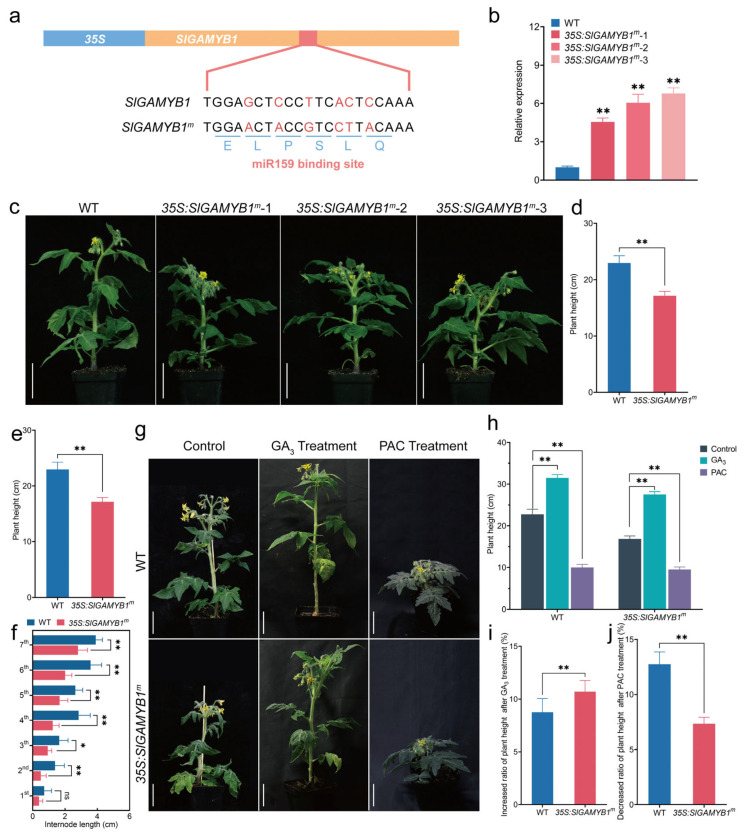
Overexpression of the Sly-miR159-resistant version of *SlGAMYB1* (*35S:SlGAMYB1^m^*) resulted in strong plant dwarfism. (**a**) Schematic representation of the *35S:SlGAMYB1* and *35S:SlGAMYB1^m^* transgenes. The red letters indicate the mutated nucleotides that alter the Sly-miR159 binding sequence in *SlGAMYB1^m^*. The encoded amino acids (ELPSLQ) are listed below the gene sequences, which show that the mutations in *SlGAMYB1^m^* do not change the protein sequence of SlGAMYB1. (**b**) Expression of *SlGAMYB1* in the leaves of wild type (WT) and *35S:SlGAMYB1^m^* at 30 days post-germination. (**c**) Phenotypes of WT and *35S:SlGAMYB1^m^* plants at anthesis. The *35S:SlGAMYB1^m^* plant exhibits a pronounced dwarfism phenotype compared to WT. (**d**) Plant height of WT and *35S:SlGAMYB1^m^* plants at anthesis. (**e**) The number of stem internodes of WT and *35S:SlGAMYB1^m^* plants at anthesis. (**f**) The length of internodes of WT and *35S:SlGAMYB1^m^* plants at anthesis. (**g**) Phenotypes of WT and *35S:SlGAMYB1^m^* plants treated with the control solution, GA_3_, and PAC. (**h**) Plant height of WT and *35S:SlGAMYB1^m^* plants treated with the control solution, GA_3_, and PAC. (**i**) The GA_3_-induced increase of plant height from control in WT and *35S:SlGAMYB1^m^* plants. (**j**) The PAC-induced decrease of plant height from the control in WT and *35S:SlGAMYB1^m^* plants. Scale bars represent 5 cm in (**c**,**g**). Error bars represent SEM (n = 3 in (**b**); n = 10 in (**d**–**f**,**h**–**j**)). Asterisks indicate a significant difference from the WT control (ns *p* > 0.05; * *p* < 0.05 and ** *p* < 0.01; Student’s *t* test in (**b**,**d**–**f**,**i**,**j**) and two-way ANOVA test in (**h**)).

To further investigate the biological functions of *SlGAMYB1* in tomato, we employed the constitutive cauliflower mosaic virus (CaMV) *35S* promoter to overexpress a Sly-miR159-resistant form of *SlGAMYB1* (*SlGAMYB1^m^*) in tomato, yielding three *35S:SlGAMYB1^m^* transgenic lines (Figure 2a). qRT-PCR confirmed a significant upregulation of *SlGAMYB1* transcript levels in these transgenic lines, with increases of 4.6-fold, 6.1-fold, and 6.8-fold relative to the wild type (Figure 2b), validating the successful overexpression of *SlGAMYB1* in the transgenic plants. The line *35S:SlGAMYB1^m^-3*, which exhibited a representative phenotype, was selected for further analysis.

Plant height is a critical agronomic trait influencing crop architecture and production strategies [28]. We measured the plant height and the internode length/number of the *35S:SlGAMYB1^m^* line in 6-week-old plants, a period marking the onset of reproductive growth. The *35S:SlGAMYB1^m^* plant displayed a pronounced dwarf phenotype compared to the wild type ‘Micro-Tom’ (Figure 2c). The wild type reached a height of 21.9 cm, while the height of the *35S:SlGAMYB1^m^* line was approximately 20% shorter (Figure 2d). In tomato, plant height is primarily determined by the internode length/number [29]. We found no difference in the number of internodes (Figure 2e); however, the internode length of the *35S:SlGAMYB1^m^* line was significantly shorter than that of the wild type (Figure 2f), suggesting that the dwarfism is primarily attributed to the inhibition of internode elongation.

**Figure 3:**
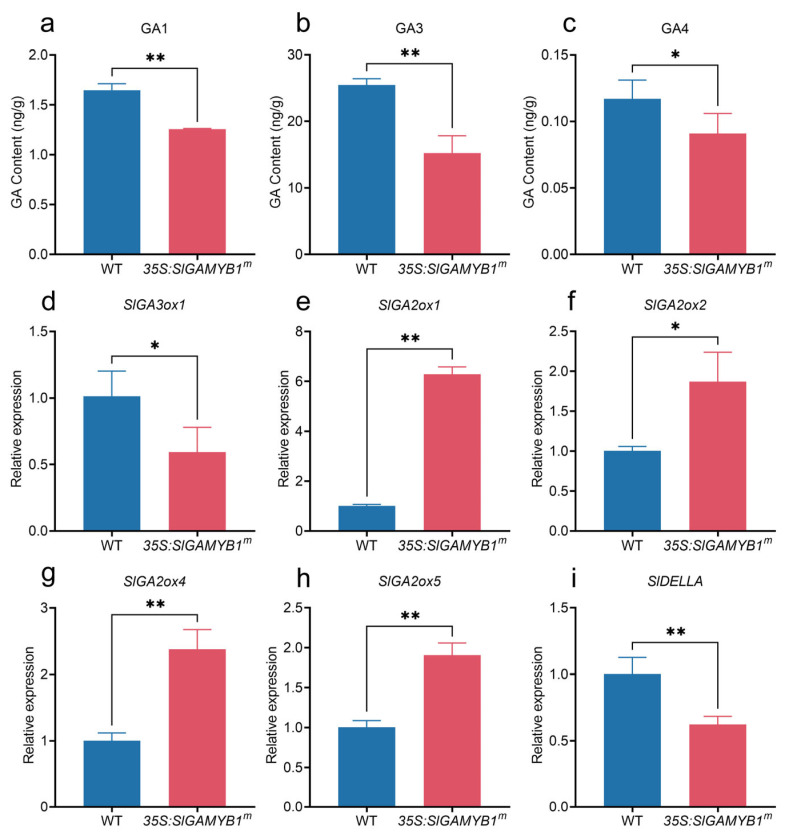
GA biosynthesis is reduced in the stem development of *35S:SlGAMYB1^m^* transgenes. (**a**–**c**) GA1 (**a**), GA3 (**b**), and GA4 (**c**) contents in the wild type (WT) and *35S:SlGAMYB1^m^* stems before anthesis. GA1, GA3, and GA4 levels are both reduced in *35S:SlGAMYB1^m^* relative to WT. (**d**–**i**) Expression levels of GA biosynthesis pathway genes in WT and *35S:SlGAMYB1^m^* stems before anthesis. The tomato *ACTIN* gene was used as the internal control. Error bars represent SEM (n = 3 in (**a**–**i**). Asterisks indicate a significant difference from the WT control (* *p* < 0.05 and ** *p* < 0.01; Student’s *t* test).

Dwarfism in plants, often associated with defects in internode elongation, has been linked to gibberellin (GA) through extensive genetic and molecular studies [30]. Our previous study identified *SlGAMYB2* as a regulator of GA biosynthesis in tomato [24]. Given the sequence similarity between SlGAMYB1 and SlGAMYB2, we hypothesized that SlGAMYB1 might affect internode elongation through GA biosynthesis. To validate this hypothesis, we examined the active GA levels in *35S:SlGAMYB1^m^* plants using liquid chromatography–mass spectroscopy (LC–MS). Comparative analysis revealed a significant reduction of GA_1_, GA_4_, and GA_3_ in *35S:SlGAMYB1^m^* lines relative to wild-type (Figure 3a–c). Moreover, we applied the effect of exogenous GA_3_ and paclobutrazol (PAC), a GA biosynthesis inhibitor, on wild-type and *35S:SlGAMYB1^m^* lines. Treatment with 100 μM exogenous GA_3_ restored the height of *SlGAMYB1*-overexpressing plants to levels comparable to the wild type (Figure 2g,h). Conversely, treatment with 100 μM PAC resulted in equal height reduction for both *35S:SlGAMYB1^m^* and wild-type plants (Figure 2g,h). The plant height in *35S:SlGAMYB1^m^* plants was more responsive to GA_3_ but less sensitive to PAC than that in wild type, as the *35S:SlGAMYB1^m^* plants showed a stronger increase and weaker decrease of plant height than wild type when treated with GA_3_ and PAC, respectively (Figure 2g–j).

**Figure A2:**
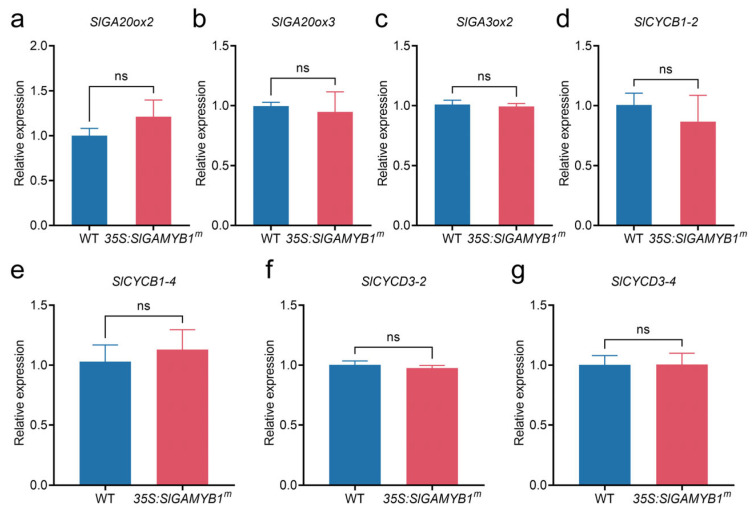
Relative expression levels of *SlGA20ox2*, *SlGA20ox3*, *SlGA3ox2*, *SlCYCB1-2*, *SlCYCB1-4*, *SlCYCD3-2* and *SlCYCD3-4* in wild type (WT) and *35S:SlGAMYB1^m^* plants. (**a**–**c**) Relative expression levels of *SlGA20ox2* (**a**), *SlGA20ox3* (**b**), and *SlGA3ox2* (**c**) in WT and *35S:SlGAMYB1^m^* stems. (**d**–**g**) Relative expression levels of *SlCYCB1-2*, *SlCYCB1-4*, *SlCYCD3-2,* and *SlCYCD3-4* in WT and *35S:SlGAMYB1^m^* leaves. Error bars represent SEM (n = 3 in (**a**–**g**)). Asterisks indicate a significant difference from the WT control (ns *p* > 0.05; Student’s *t* test), where ns indicates no significant difference between the groups.

To identify the potential target genes of SlGAMYB1, we further examined the transcription level of representative genes involved in the GA biosynthesis pathway. The transcripts of *SlGA3ox1* decreased significantly in *35S:SlGAMYB1^m^* lines, while the transcripts of *SlGA2ox1*, *SlGA2ox2*, *SlGA2ox4*, and *SlGA2ox5* increased significantly (Figure 3d–h). Neither of the two GA20 oxidase genes examined showed significant expression changes in the *35S:SlGAMYB1^m^* lines (Figure A2a–c). These findings indicated that SlGAMYB1 affects internode elongation through GA biosynthesis rather than the GA signaling pathway.

### 2.3. SlGAMYB1 Plays a Crucial Role in Controlling Leaf Morphology and Cell Characteristics in Tomato

**Figure 4:**
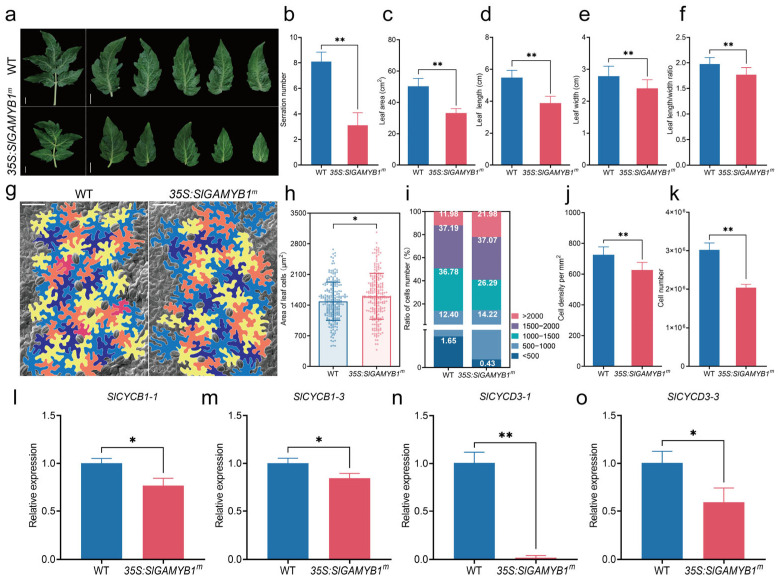
Overexpression of *SlGAMYB1* affected the leaf morphology and cell characteristics in tomato. (**a**) Phenotypic appearance of leaves from wild type (WT) and *35S:SlGAMYB1^m^* transgenic lines. (**b**) Quantification of serration number on leaves, indicating a significant decrease in *35S:SlGAMYB1^m^* compared to WT. (**c**) Measurement of leaf area, demonstrating a smaller leaf area in *35S:SlGAMYB1^m^* compared to WT. (**d**) Comparison of leaf length, with *35S:SlGAMYB1^m^* plants exhibiting shorter leaves than WT. (**e**) Comparison of leaf width, with *35S:SlGAMYB1^m^* plants exhibiting shorter leaves than WT. (**f**) The leaf length-to-width ratio is reduced in *35S:SlGAMYB1^m^* plants compared to WT, suggesting changes in leaf shape. (**g**) Scanning Electron Micrographs (SEM) of leaf epidermal cells from both WT and *35S:SlGAMYB1^m^* plants, illustrating the differences in cell size and morphology. (**h**) Scatter plot of cell size distribution, indicating a significant increase in larger cell sizes in *35S:SlGAMYB1^m^* plants compared to WT. (**i**) Bar chart representing the ratio of cell numbers across different size categories, showing a higher proportion of larger cells in *35S:SlGAMYB1^m^* plants compared to WT. (**j**) Measurement of cell density per mm^2^, which is reduced in *35S:SlGAMYB1^m^* plants compared to WT. (**k**) Quantification of total cell number, with *35S:SlGAMYB1^m^* plants having a significantly lower cell count than WT. (**l**–**o**) Relative expression levels of *SlCYCB1-1* (**l**), *SlCYCB1*-3 (**m**), *SlCYCD3-1* (**n**) and *SlCYCD3-3 *(**o**) in WT and *35S:SlGAMYB1^m^* leaves. Both genes show a downregulation in the *35S:SlGAMYB1^m^* line, with *SlCYCD3-1* being more significantly reduced than *SlCYCD3-3*, highlighting the differential impact on cell cycle progression. Scale bars represent 1 cm in (**a**) and 50 μm in (**g**). Error bars represent SEM (n = 10 in (**b**–**f**,**h**–**k**); n = 3 in (**l**–**o**)). Asterisks indicate a significant difference from the WT control (* *p* < 0.05 and ** *p* < 0.01; Student’s *t* test).

Tomato compound leaves comprise a terminal lobed leaflet with two pairs of lateral leaflets, separated by a rachis (Figure 4a). The *35S:SlGAMYB1^m^* plants exhibited a visible pleiotropic phenotype in leaf morphology, including changes in the serration of leaf margins and overall leaf dimensions (Figure 4a). Notably, the number of serrations was significantly reduced in *35S:SlGAMYB1^m^* lines compared to the wild type (Figure 4b). Quantitative analysis of the area, length, and width of mature leaves revealed that the leaf area of *35S:SlGAMYB1^m^* was reduced compared to the wild type. The results showed that *35S:SlGAMYB1^m^* had a decreased area relative to wild type (Figure 4c). Further detailed measurement revealed that both leaf length and width were considerably decreased in *35S:SlGAMYB1^m^* (Figure 4d,e), suggesting that the reduction in leaf area was primarily attributed to the diminished leaf dimensions. Moreover, the leaf length-to-width ratio, a critical parameter influencing leaf photosynthetic capacity and overall plant architecture, was notably reduced in *35S:SlGAMYB1^m^* plants, highlighting the role of SlGAMYB1 in leaf morphogenesis. (Figure 4f). This change in leaf morphology might be attributed to the role of SlGAMYB1 in regulating cell division, elongation, or differentiation processes during leaf ontogeny.

In the process of leaf morphogenesis, precise control of cell proliferation and growth is crucial for generating different leaf sizes and shapes [31]. Subsequently, we analyzed the cell number and size in mature leaves of the *35S:SlGAMYB1^m^* and wild type lines using scanning electron microscopy (SEM) (Figure 4g). Our findings showed that the leaf epidermal cells of *35S:SlGAMYB1^m^* were larger than those of the wild type (Figure 4h). A comprehensive assessment of cellular composition was achieved by comparing the total cell count with the distribution of leaf epidermal cells of varying sizes (Figure 4g). The proportion of large cells (with an area over 2000 μm^2^) was higher in *35S:SlGAMYB1^m^* plants, while the number of small cells (with an area under 500 μm^2^) was significantly reduced (Figure 4i, Appendix A Table A1). However, both cellular density and total cell count were lower in *35S:SlGAMYB1^m^* leaves, indicating a decrease in total cell numbers and suggesting that cell proliferation was inhibited while cell expansion was promoted (Figure 4j,k).

Leaf development is intricately regulated by the cell cycle machinery, with B-type and D-type cyclins playing crucial roles in controlling cell division and expansion, essential for plant growth and development [32,33]. Our molecular analysis uncovered significant alterations in their expression profiles in the developing leaves (Figure 4i–o and Figure A2d–g). Specifically, the expression levels of key cell cycle-related genes [34], including *SlCYCB1-1*, *SlCYCB1-3*, *SlCYCD3-1*, and *SlCYCD3-3*, were substantially downregulated in *35S:SlGAMYB1^m^* plants compared to wild type (Figure 4i–o). Notably, *SlCYCB1-1*, *SlCYCB1-3,* and *SlCYCD3-3* exhibited a decrease in expression that reached statistical significance (* *p* < 0.05), while *SlCYCD3-1* displayed an even more pronounced decrease (** *p* < 0.01). The downregulation of cell cycle-related genes, highlighting the potential role of SlGAMYB1, interferes with normal cell cycle progression, potentially by promoting cell cycle arrest or senescence.

### 2.4. Overexpression of SlGAMYB1 in Tomato Promotes Flowering and Leads to an Increased Number of Floral Organs

**Figure 5:**
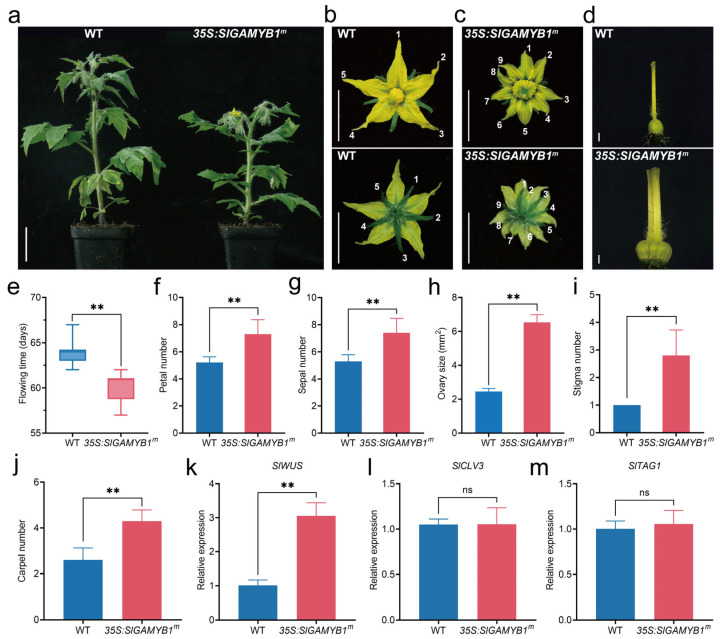
Overexpression of *SlGAMYB1* promotes early flowering and enhances floral organogenesis. (**a**) Comparative flowering phenotypes between wild type (WT) and *35S:SlGAMYB1^m^* plants, showcasing the advanced flowering in *35S:SlGAMYB1^m^* lines. (**b**,**c**) Detailed floral phenotypes of WT and *35S:SlGAMYB1^m^* plants, highlighting the morphological differences in flower structure. (**d**) Ovary phenotypes of WT and *35S:SlGAMYB1^m^* plants, with a focus on the size and shape of the ovaries. (**e**) Quantitative analysis of flowering time, demonstrating a significant reduction in the days to flowering in *35S:SlGAMYB1^m^* compared to WT. (**f**–**j**) Analysis of floral organ number and size, including petal number (**f**), sepal number (**g**), ovary size (**h**), stigma number (**i**), and carpel number (**j**), demonstrating an increase in both the quantity and dimensions of floral organs in *35S:SlGAMYB1^m^* compared to WT. (**k**–**m**) Relative expression levels of *SlWUS* (*tomato WUSCHEL homolog*) (**k**), *SlCLV3* (*CLAVATA3 homolog*) (**l**), and *SlTAG1* (*TOMATO AGAMOUS-LIKE 1*) (**m**) in WT and *35S:SlGAMYB1^m^* shoot apical meristem (SAM). Scale bars represent 5 cm in (**a**), 1 cm in (**b**,**c**), and 1 mm in (**d**). Error bars represent SEM (n = 10 in (**e**–**j**); n = 3 in (**k**–**m**)). Numerals (1–9) denoting petal/sepal numbers per flower. Asterisks indicate a significant difference from the WT control (ns *p* > 0.05 and ** *p* < 0.01; Student’s *t* test).

Previous studies highlighted the potential effect of GAMYB on flowering [4]; therefore, the flowering time in the T1 generation of *35S:SlGAMYB1^m^* lines was investigated. Our observations revealed that the first flower opened at 57–62 days after sowing in the progenies of *35S:SlGAMYB1^m^*, preceding the 63–67 days observed in wild-type plants (Figure 5e). Moreover, we counted the number of leaves produced before the first flower appeared in *35S:SlGAMYB1^m^* compared with wild-type plants and found that flowering initiated in *35S:SlGAMYB1^m^* after the emergence of six leaves, whereas the wild type formed seven leaves at flowering (Figure 5f). These findings suggest that SlGAMYB1 can accelerate the transition to flowering in tomato.

In addition to the significant impact on vegetative growth, overexpression of *SlGAMYB1* also exerts a profound influence on reproductive development, particularly in the formation of floral organs. To characterize the impact on flower development in *35S:SlGAMYB1^m^* plants, we quantified the number of floral organs at the anthesis stage. The wild type flowers are composed of 5–6 green sepals, alternating with a similar number of yellow petals, about 5–6 yellow stamens forming a staminal cone around the pistil, and 2–3 fused carpels (Figure 5b–d). In contrast, the flowers of *35S:SlGAMYB1^m^* had 7–9 sepals, 7–9 petals, 8–10 stamens, and 4–6 carpels (Figure 5b–d). The marked increase in the number of petals, stamens, and carpels in *35S:SlGAMYB1^m^* compared to the wild type underscores the pivotal role of *SlGAMYB1* in controlling organ number during flower development in tomato.

**Figure A3:**
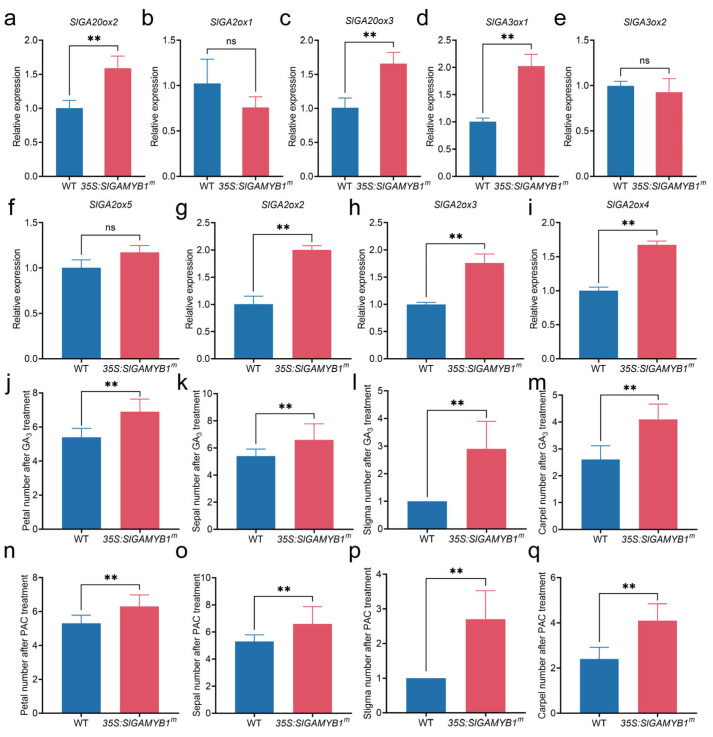
SlGAMYB1 regulates floral organ number independently of gibberellin (GA) signaling. (**a**–**i**) Expression levels of GA biosynthesis pathway genes in WT and *35S:SlGAMYB1^m^* meristem. The tomato *ACTIN* gene was used as the internal control. (**j**–**m**) Effects of GA_3_ treatments on petal number (**j**), sepal number (**k**), stigma number (**l**), and carpel number (**m**) in WT and *35S:SlGAMYB1^m^* plants. (**n**–**q**) Effects of PAC treatments on petal number (**n**), sepal number (**o**), stigma number (**p**), and carpel number (**q**) in WT and *35S:SlGAMYB1^m^* plants. Error bars represent SEM (n = 3 in (**a**–**q**)). Asterisks indicate a significant difference from the WT control (ns *p* > 0.05 and ** *p* < 0.01; Student’s *t* test).

To determine whether the observed floral organ phenotype is mediated by changes in gibberellin (GA) levels, we treated *35S:SlGAMYB1^m^* and wild-type plants with exogenous GA and PAC prior to floral primordium formation. Surprisingly, neither treatment induced significant changes in floral organ number in either genotype (Figure A3), suggesting that *SlGAMYB1*-mediated floral organ proliferation is largely GA-independent. However, qRT-PCR analysis revealed that *35S:SlGAMYB1^m^* plants exhibit concurrent upregulation of both GA biosynthesis genes (*GA20ox*, *GA3ox*) and the GA catabolism gene (*GA2ox*) (Figure A3). This paradoxical transcriptional response implies that *SlGAMYB1* may regulate GA homeostasis rather than simply promoting GA accumulation.

Previous studies have established that mutations affecting floral organ number and fruit locule formation (e.g., *fas*, *lc*, and *eno*) are consistently associated with an enlarged shoot apical meristem (SAM) [35,36]. This correlation strongly suggests that SAM development serves as a critical determinant of organ differentiation patterns in plants, a principle well-documented in Arabidopsis [37,38,39,40]. To investigate whether *SlGAMYB1* overexpression influences SAM development in tomato, we analyzed the expression profiles of three key SAM regulatory genes—*SlWUS* (a promoter of stem cell maintenance), *SlCLV3* (a negative regulator of SAM size), and *SlTAG1* (a marker of floral meristem identity)—in *35S:SlGAMYB1^m^* lines. Quantitative RT-PCR revealed a striking upregulation of *SlWUS* (three-fold increase) in *35S:SlGAMYB1^m^* plants compared to wild-type plants (Figure 5k). In contrast, the expression levels of *SlCLV3* and *SlTAG1* remained statistically unchanged (Figure 5l,m), indicating that SlGAMYB1 specifically targets the *SlWUS*-mediated pathway. This transcriptional shift mirrors the molecular phenotypes observed in *fas* and *lc* mutants, where *WUS* overexpression leads to SAM expansion and subsequent ectopic organ formation [35,37].

## 3. Discussion

### 3.1. Evolutionary Conservation and Functional Diversification of SlGAMYB1 in Tomato

The high level of sequence similarity across the R2R3 DNA-binding domains of GAMYB proteins from various species (Figure 1b) highlights the essential nature of this domain in the structural and functional aspects of these proteins. This conservation reflects a deep evolutionary connection, suggesting a shared ancestry and potentially analogous regulatory roles in plant development and responses to environmental cues. This serves as a testament to the R2R3 domain’s critical importance in the transcriptional machinery of plants, where it likely mediates gene expression in response to various developmental and stress signals.

The distinct branching of GAMYB proteins in the phylogenetic analysis (Figure 1c) implies a potential functional diversification of GAMYB genes within different species. The aggregation of GAMYB proteins from Solanaceae family members into a singular group hints at a shared ancestral lineage and potentially overlapping biological functions. This distinct grouping reinforces their taxonomic relationships and provides insights into their potential roles in the evolutionary trajectory of plant development and adaptation.

The tissue-specific expression dynamics of *SlGAMYB1* (Figure 1d), with pronounced expression in reproductive tissues, suggest its role as a regulatory hub in developmental processes, particularly during the reproductive phase in tomato. The significant upregulation of *SlGAMYB1* expression during the reproductive phase mirrors the expression profile of *SlGAMYB2*, suggesting potential parallelism in their regulatory functions [4]. This concordance posits *SlGAMYB1* as a significant player in the ontogenetic development of both vegetative and reproductive structures, with a specialized role in the intricate processes underlying floral development.

The coordinated expression of these GAMYB genes may be integral to the precise orchestration of developmental transitions, particularly those leading to the formation and maturation of floral organs. The implications of SlGAMYB1 in these processes are further underscored by its potential to modulate gene networks that are responsive to both endogenous and environmental cues, thereby fine-tuning the floral developmental program in tomato. This study provides a comprehensive analysis of SlGAMYB1, highlighting its potential as a key regulator in tomato development and laying the groundwork for future research into its functional significance in shaping plant development and adaptation.

### 3.2. SlGAMYB1 Orchestrates GA Homeostasis to Fine-Tune Plant Stature in Tomato

Our study provides evidence that SlGAMYB1 plays a crucial role in regulating plant height by modulating GA metabolism. The *35S:SlGAMYB1^m^* plant presents a dwarf phenotype characterized by reduced plant height and internode length, which is primarily attributed to the inhibition of internode elongation (Figure 2c–f). This phenotype is associated with significant reductions in active GA levels, including GA_1_, GA_4_, and GA_3_ (Figure 3a–c), which are well-known for their roles in promoting internode elongation and plant height [41,42].

The molecular analysis of *35S:SlGAMYB1^m^* lines reveals a coordinated transcriptional reprogramming of GA metabolic genes. Specifically, SlGAMYB1 suppresses the expression of *SlGA3ox1*, which catalyzes the final step in the synthesis of bioactive GAs, leading to reduced accumulation of GA_1_, GA_4_, and GA_3_ [43]. Concurrently, SlGAMYB1 activates the expression of GA catabolism genes (*SlGA2ox1/2/4/5*), enhancing the degradation of bioactive GAs. This dual regulatory mode ensures a robust reduction in bioactive GA levels, consistent with the severe dwarfism observed in *35S:SlGAMYB1^m^* plants.

The findings that SlGAMYB1 directly modulates GA biosynthesis and catabolism genes highlight its role as a key transcriptional modulator of GA flux. This regulation ultimately restricts stem elongation, leading to the observed dwarf phenotype. Such bidirectional regulation—curtailing synthesis while accelerating turnover—ensures a robust reduction in bioactive GA levels, consistent with the severe dwarfism observed. Similar mechanisms have been reported in rice, where OsGAMYB suppresses *GA3ox2* while inducing *GA2ox3* [44], though the specific *GA2ox* isoforms targeted by SlGAMYB1 in tomato highlight species-specific regulatory divergence.

The downregulation of *SlDELLA* transcripts in *35S:SlGAMYB1^m^* plants (Figure 3i), despite reduced GA levels, presents an intriguing paradox. This suggests that SlGAMYB1 may directly suppress *SlDELLA* expression or activate compensatory growth-restricting pathways. The persistence of dwarfism despite *SlDELLA* downregulation implies the possibility of residual DELLA protein activity, phosphorylation modification of DELLA protein, GA-independent growth limitation, or alternative repressors dominating the phenotype [45,46,47]. These findings reveal a non-canonical layer of GA-height regulation, where SlGAMYB1 orchestrates both GA metabolism and *DELLA* transcription to fine-tune plant stature.

Understanding the molecular mechanisms by which SlGAMYB1 regulates GA metabolism and plant growth could provide valuable insights for breeding efforts aimed at modulating plant architecture and improving crop yield. Future studies should focus on elucidating the direct interactions between SlGAMYB1 and its target genes, as well as the broader implications of its regulatory role in plant development and adaptation to environmental cues.

### 3.3. SlGAMYB1 Modulates Leaf Development Through Differential Regulation of Cyclin-Dependent Cell Cycle Progression

Our study provides evidence that SlGAMYB1 exerts a significant influence on leaf development by modulating the expression of critical cell cycle genes, particularly D-type cyclins (Figure 4). The more pronounced effect on cell number compared to cell size underscores the importance of D-type cyclins in controlling cell proliferation [32,33]. This finding is significant as it highlights the potential of SlGAMYB1 as a regulatory factor in determining leaf morphology and cell characteristics.

B-type cyclins, primarily responsible for managing cell division during the G1/S transitions, experienced a slight downregulation in *35S:SlGAMYB1^m^* plants (Figure 4l,m), hinting at possible disruptions in cell division that could result in larger cell sizes due to prolonged cell expansion. This impact on cell size may also be indirect, as these cyclins regulate the timing of endoreduplication entry, further affecting cell dimensions [48,49]. In contrast, D-type cyclins, essential for promoting cell proliferation during the G1 phase by enabling the G1-to-S transition [50,51], showed significant downregulation of *SlCYCD3-1* in *35S:SlGAMYB1^m^* plants (Figure 4n), indicating a stronger influence on cell proliferation. The direct involvement of D-type cyclins in initiating DNA replication and cell proliferation makes them pivotal in controlling cell number, thus highlighting their critical role in determining leaf cell count. The more significant downregulation of D-type cyclins compared to B-type cyclins in *35S:SlGAMYB1^m^* plants implies that SlGAMYB1 has a greater impact on cell number than on cell size. This could be due to the direct role of D-type cyclins in promoting cell proliferation, which is a more critical determinant of cell number than cell size. The downregulation of D-type cyclins by SlGAMYB1 could lead to a reduction in the number of cells entering the S phase, thereby affecting cell proliferation and contributing to the observed decrease in cell number per leaf.

### 3.4. SlGAMYB1 Promotes Floral Organogenesis Through WUS-Mediated Shoot Apical Meristem Regulation Independent of GA Signaling

The overexpression of *SlGAMYB1* in tomato significantly promotes flowering and increases the number of floral organs (Figure 5a–j), suggesting a crucial role in reproductive development. The acceleration of flowering and the increase in floral organ number in *35S:SlGAMYB1^m^* plants indicate that SlGAMYB1 may regulate floral organogenesis by influencing the shoot apical meristem (SAM) [35,36]. The upregulation of *SlWUS* (Figure 5k), a key regulator of stem cell maintenance, in *35S:SlGAMYB1^m^* plants suggests that SlGAMYB1 may enhance stem cell proliferation, leading to an enlarged SAM and increased floral organ number [37,38,39,40].

The lack of significant changes in floral organ number upon GA and PAC treatment (Figure A3) implies that SlGAMYB1-mediated effects on floral organ proliferation are largely independent of GA signaling. This is further supported by the concurrent upregulation of both GA biosynthesis and catabolism genes in *35S:SlGAMYB1^m^* plants, indicating that SlGAMYB1 may modulate GA homeostasis rather than simply promoting GA accumulation.

The selective upregulation of *SlWUS* in *35S:SlGAMYB1^m^* lines provides mechanistic insight into how *SlGAMYB1* may influence fruit morphology. As a central regulator of stem cell proliferation, *WUS* is known to establish a positive feedback loop with *CLV3* to maintain SAM homeostasis [38,39]. Our finding that *SlCLV3* expression remains unaltered despite *SlWUS* induction suggests two non-exclusive possibilities: *SlGAMYB1* may bypass the canonical WUS-CLV3 feedback loop, potentially through direct transcriptional activation of *SlWUS*, or the temporal dynamics of *CLV3* repression may be delayed relative to *WUS* activation, as reported in *Arabidopsis* inflorescence meristems [52].

The phenotypic parallels between *35S:SlGAMYB1^m^* plants and *fas/lc* mutants further support the hypothesis that *SlGAMYB1* acts upstream of SAM size determination. Notably, the lack of *SlTAG1* expression changes implies that *SlGAMYB1*-mediated effects are distinct from pathways governing floral meristem identity, instead focusing on stem cell pool expansion. This specificity aligns with studies showing that *WUS* overexpression alone is sufficient to drive multilocular fruit formation without altering floral organ identity genes [36]. The uncoupling of *SlWUS* activation from *SlCLV3* repression could confer developmental plasticity under environmental stresses where transient SAM expansion is advantageous. From a breeding perspective, targeted manipulation of *SlGAMYB1* expression might offer a tunable strategy to modulate locule number—a key yield-associated trait—without pleiotropic effects on floral architecture. Future work should explore whether *SlGAMYB1* physically interacts with the *SlWUS* promoter and how this pathway integrates with hormonal signals (e.g., auxin) known to regulate SAM activity.

## 4. Materials and Methods

### 4.1. Sequence Alignment and Phylogenetic Analysis

The amino acid sequences of related GAMYB proteins in various species were obtained from the Solanaceae Genomics Network (http://www.solgenomics.net) and National Center for Biotechnology Information (http://www.ncbi.nlm.nih.gov) database (Appendix A Table A3). Then, multiple-sequence alignment was carried out using MEGA11 software, and boxes highlighting conserved sequences were drawn using the online software ESPript 3.0 “https://espript.ibcp.fr/ESPript/cgi-bin/ESPript.cgi (accessed on 17 November 2024)”. The phylogenetic analysis was conducted via the neighbor-joining method with MEGA11, and bootstrapping was performed with 1000 replications.

### 4.2. Plant Materials and Growth Conditions

Tomato (*Solanum lycopersicum cv* Micro-Tom) was used in this study. The seeds were germinated on wet filter paper in a petri dish at 28 °C in the dark for 2 days. Then the resulting seedlings were grown in a greenhouse under a 16-h light, 8-h dark photoperiod with temperatures of 25 °C/18 °C in day/night. Water management and pest control were meticulously executed in accordance with established protocols.

### 4.3. Vector Construction and Plant Transformation

To generate *35S:SlGAMYB1^m^*, the coding region sequence of a Sly-miR159-resistant form of *SlGAMYB1* was amplified using primers containing *BamH I* and *Sal I* sites and then inserted into the *PBI121* vector. Then the construct was introduced in tomato (*Solanum lycopersicum cv* Micro-Tom) using the *Agrobacterium tumefaciens*-mediated transformation [53]. The presence of the transgene in each transgenic line was verified in the first generation of transformation (T0) using PCR. Furthermore, the increased expression of *SlGAMYB1* was confirmed in *35S:SlGAMYB1^m^* transgenic lines using qRT-PCR. Primers for PCR and qRT-PCR analyses are listed in Appendix A Table A2.

### 4.4. RNA Extraction and Quantitative Real-Time PCR (qRT-PCR) Analysis

Total RNA was extracted from various tissues of wild type and different transgenic tomato plants (details in the results and figures) using Trizol reagent (Thermo Scientific, Waltham, MA, USA) according to the manufacturer’s protocol. The quantity and quality of RNA were estimated using a NanoDrop 2000 spectrophotometer (Thermo Scientific, Waltham, MA, USA). After removing genomic DNA with DNaseI, the first-strand cDNA was synthesized using a TAKARA first-strand cDNA synthesis kit (TaKaRa, Japan). Quantitative real-time PCR (qRT-PCR) was performed with SYBR Green detection on Bio-Rad CFX96 (Bio-Rad, Hercules, CA, USA). Relative gene expression was analyzed using the 2^−ΔΔCt^ method [54] from three biological replicates. Tomato *ACTIN* (*Solyc11g005330*) was used as the internal control for quantitation of mRNA. Primers used for reverse transcription and qRT-PCR are listed in Appendix A Table A2.

For the evaluation of reference gene stability assay, four candidate reference genes (*ACTIN*, *UBI*, *EF-1α*, and *TUB*) were selected using the BestKeeper algorithm [27]. qRT-PCR was performed in triplicate on a Bio-Rad CFX96 system with SYBR Green Master Mix. The amplification efficiency (90–110%) and specificity of each primer pair (Appendix A Table A2) were validated via standard curves and melt curve analysis. BestKeeper calculates gene stability based on the standard deviation (SD) and coefficient of variation (CV) of cycle threshold (Ct) values across samples. Lower SD/CV values indicate higher stability. Among the tested genes, *ACTIN* exhibited the highest stability (BestKeeper index = 0.657), followed by *UBI*, *EF-1α*, and *TUB*. Consequently, *ACTIN* was selected as the internal control for subsequent qRT-PCR analyses.

### 4.5. Plant Height Analyses

To characterize the dwarf phenotype, plant height, internode number, and internode length were measured for 6-week-old seedlings of all the lines (*35S:SlGAMYB1^m^* and wild type). Each line was represented by nine plants to ensure statistical reliability. Plant height was determined by measuring the vertical distance from the soil surface to the shoot apex. The internode count was initiated by designating the first internode as the one situated between the cotyledons and the first true leaf of the main stem. The number of internodes was recorded from the cotyledon to the top of the main stem. Additionally, the length of each individual internode was meticulously recorded to provide a detailed analysis of the plant’s growth pattern. Plant height was measured using digital calipers (Mitutoyo, ±0.01 mm accuracy), with three technical replicates per measurement.

### 4.6. Gibberellin Quantification and GA3/Paclobutrazol (PAC) Treatment

About 1 g of stems from wild type and *35S:SlGAMYB1^m^* were harvested before anthesis (30 days after seed germination) and sent to the facilities in Wuhan Metware Biotechnology Co., Ltd. (Wuhan, 430070, China) for GA quantification. GAs were extracted from three independent pools of ovaries and analyzed based on the published protocols using a mass spectrometer [55,56].

For the gibberellic acid (GA_3_) and paclobutrazol (PAC) treatments, a 10 mL solution of 0.1 mM GA_3_, 0.1 mM PAC (with a purity of ≥95.0%, sourced from Sigma-Aldrich, Darmstadt, Germany), or a control solution consisting of 0.095% ethanol, was applied directly to the roots of 15-day-old plants (n = 10 per treatment group, randomized block design). This treatment was administered every ten days, with regular watering maintained throughout the interim periods. Three independent biological replicates were performed (total N = 30 plants per treatment across replicates). Plant height was measured using digital calipers (Mitutoyo, ±0.01 mm accuracy), with three technical replicates per measurement. Floral organ counts were conducted at the anthesis stage (n = 10 flowers per treatment group, randomly selected).

### 4.7. Scanning Electron Microscopy (SEM)

The transition zones of mature leaves were prepared for SEM observation as described [57]. Briefly, the transition zones were hand-dissected and fixed in 2.5% (*w*/*v*) glutaraldehyde in 0.1-M cacodylate (pH 7.4) buffer and stored in 70% ethanol. Subsequently, tissue dehydration was carried out through a graded ethanol series, escalating from 70% to 100%, after which the samples were subjected to critical point drying using a Bal-Tec CPD030 apparatus (Leica Microsystems, Wetzlar, Germany). This process involved the substitution of water with liquid CO_2_, which was subsequently evaporated at the critical point for CO_2_, effectively removing all liquid without causing damage to the sample’s morphology. Following dehydration, the samples underwent gold coating in a Bal-Tec SCD005 Sputter Coater (BalTec, Pfäffikon, Switzerland) to enhance conductivity and resolution. The coated samples were then examined using a Hitachi S-3500N scanning electron microscope (Hitachi, Tokyo, Japan) operated at an acceleration voltage of 10 kV. For quantitative analysis, the dimensions and number of leaf cells were determined using the ImageJ (Version 1.46r) software (https://imagej.net/ij/). Cells were categorized by size, and their distribution was determined relative to total cell counts. Cell density was calculated by dividing the total number of cells by the measured leaf area. Statistical analysis was performed using a Student’s *t* test to assess significance, with data presented as mean ± standard error from three biological replicates.

## 5. Conclusions

This study elucidates the diverse regulatory roles of SlGAMYB1 in tomato development, demonstrating its dual impact on vegetative and reproductive processes. Through GA-mediated pathways, SlGAMYB1 controls plant architecture by reducing height and internode length while simultaneously regulating leaf morphology via cell cycle modulation. Significantly, SlGAMYB1 enhances floral organ number and accelerates flowering through WUS-mediated SAM expansion, independent of GA signaling. These findings establish SlGAMYB1 as a central coordinator of developmental transitions, offering novel genetic targets for improving tomato architecture and yield potential through molecular breeding strategies.

## Data Availability

The original contributions presented in this study are included in the article. Further inquiries can be directed to the corresponding author.

## Abbreviations

The following abbreviations are used in this manuscript:

GA	Gibberellin
PAC	Paclobutrazol
SEM	Scanning electron microscopy
qRT-PCR	Quantitative real-time PCR
GA_1_/_3_/_4_	Gibberellic acid (bioactive GA form)
LC-MS	Liquid chromatography–mass spectrometry
ORF	Open reading frame
UTR	Untranslated region
NJ	Neighbor-joining
SAM	Shoot apical meristem
SEM	Standard error of the mean
ANOVA	Analysis of variance

## Appendix A

**Table A1:** The proportion of cells of different sizes.

Different Sizes of Cell	WT	*35S:SlGAMYB1^m^*
<500 μm^2^	1.65%	0.43%
500–1000 μm^2^	12.40%	14.22%

**Table A2:** List of primers used in this study.

Gene	Primer Name	Sequence	Purpose
*SlGAMYB1*	SlGAMYB1-qF	GAGATTAAGCAAGAGATTGGCG	qRT-PCR
*SlGAMYB1*	SlGAMYB1-qR	AACCAAAAGGATCTTTCGAAGC	qRT-PCR
*Actin*	Actin-F	TGTTGCTATTCAGGCTGTGC	qRT-PCR
*Actin*	Actin-R	CTGCTCCTGGCAGTTTCAAT	qRT-PCR
*SlGAMYB1*	PBI-SlGAMYB1m-F1	ATGAGCATCAAAAGTGAAACC	Overexpression
*SlGAMYB1*	PBI-SlGAMYB1m-R1	TCATAATCTCATTCTTCCTGTTG	Overexpression
*SlGAMYB1*	PBI-SlGAMYB1m-F2	CAGAGCCCACATGGGCAATGAAGCTGGAACTACCGTCCTTACAAAACCAGACAGAGAAC	Overexpression
*SlGAMYB1*	PBI-SlGAMYB1m-R2	TAAGGACGGTAGTTCCAGCTTCATTGCCCATGTGGGCTCTGAAGAGGAGTTGCCATTTA	Overexpression
*SlGAMYB1*	SlGAMYB1-qF	GAGATTAAGCAAGAGATTGGCG	qRT-PCR
*TUB*	TUB-F	TTGGTTTTGCACCACTGACTTC	qRT-PCR
*TUB*	TUB-R	AAGCTCTGGCACTGTCAAAGC	qRT-PCR
*EF-1* *α*	EF-1α-F	ATTGGAAATGGATATGCTCCA	qRT-PCR
*EF-1* *α*	EF-1α-R	TCCTTACCTGAACGCCTGTCA	qRT-PCR
*UBI*	UBI-F	TCGTAAGGAGTGCCCTAATGCTGA	qRT-PCR
*UBI*	UBI-R	CAATCGCCTCCAGCCTTGTTGTAA	qRT-PCR
*SlCycB1-1*	SlCycB1-1-F	CTGGTTTCTCAGAGTCTCAAGT	qRT-PCR
*SlCycB1-1*	SlCycB1-1-R	ACCTTAAGCTTGTGATTTGCAG	qRT-PCR
*SlCycB1-2*	SlCycB1-2-F	GGACAGTTGGAGTGGTACTTAA	qRT-PCR
*SlCycB1-2*	SlCycB1-2-R	GCATAATTCATCAGCCCCAATT	qRT-PCR
*SlCycB1-3*	SlCycB1-3-F	GGCTGATGAACTACACTACTGT	qRT-PCR
*SlCycB1-3*	SlCycB1-3-R	AACTTCCTATAAACCGCCTTCA	qRT-PCR
*SlCycB1-4*	SlCycB1-4-F	GCTGCGGATGTTGATAATCATT	qRT-PCR
*SlCycB1-4*	SlCycB1-4-R	TGTAGTCATTCACTCGACCTTC	qRT-PCR
*SlCycD3-1*	SlCycD3-1-F	CTGTTTTTGAGAATCGAGTCCG	qRT-PCR
*SlCycD3-1*	SlCycD3-1-R	TCATCCTCTAACAAATCACCCC	qRT-PCR
*SlCycD3-2*	SlCycD3-2-F	CTCTGCTCAAACTGCAATTCTT	qRT-PCR
*SlCycD3-2*	SlCycD3-2-R	TGGGTCTCTTCAATTTTTGCAG	qRT-PCR
*SlCycD3-3*	SlCycD3-3-F	GGAAGAAGAAGAACTTACCTCTCT	qRT-PCR
*SlCycD3-3*	SlCycD3-3-R	ACTGCAAGAAATCCAGTTTGAG	qRT-PCR
*SlCycD3-4*	SlCycD3-4-F	TACTGCTACCACTGCTGTTTTA	qRT-PCR
*SlCycD3-4*	SlCycD3-4-R	CTGACTCATCCAAGGCTTATCT	qRT-PCR
*SlCycD3-5*	SlCycD3-5-F	ATGTGACATGTTCTGGGAAGAT	qRT-PCR
*SlCycD3-5*	SlCycD3-5-R	CTAACACAGCAGTCAAAGCATT	qRT-PCR
*SlCycD3-6*	SlCycD3-6-F	CCAAAGTATGTGTTTGAGGCAA	qRT-PCR
*SlCycD3-6*	SlCycD3-6-R	GGTGTCACTGGATTCATTTTCC	qRT-PCR
*SlCycD3-7*	SlCycD3-7-F	AGATGAGGGAGATTTGGGAGGA	qRT-PCR
*SlCycD3-7*	SlCycD3-7-R	TCTTAACCCCAACAAAACCCCA	qRT-PCR
*S* *l* *GA20ox2*	RT-SlGA20ox2-F	GTGATCCGATTGCAGCTAAGCG	qRT-PCR
*S* *l* *GA20ox2*	RT-SlGA20ox2-R	ACCGATGTGCAAGTGAGATAAG	qRT-PCR
*S* *l* *GA20ox3*	RT-SlGA20ox3-F	CTAGTGTTACTAGAGAACTACA	qRT-PCR
*S* *l* *GA20ox3*	RT-SlGA20ox3-R	TGTCAACCCATGGTTAACCAC	qRT-PCR
*S* *l* *GA3ox1*	RT-SlGA3ox1-F	GAATCCCATGCATGGAATCAT	qRT-PCR
*S* *l* *GA3ox1*	RT-SlGA3ox1-R	TGTTATCGAGGTCGATCACTGG	qRT-PCR
*S* *l* *GA3ox2*	RT-SlGA3ox2-F	ATTGGACGACGATGGATCGCG	qRT-PCR
*S* *l* *GA3ox2*	RT-SlGA3ox2-R	GCATGCATGGCCAATTGTATCC	qRT-PCR
*S* *l* *GA2ox1*	RT-SlGA2ox1-F	CATAGTGAAAGCCTCTGAAG	qRT-PCR
*S* *l* *GA2ox1*	RT-SlGA2ox1-R	CAACTTCACCATTATCTCCA	qRT-PCR
*S* *l* *GA2ox2*	RT-SlGA2ox2-F	CTCATCGTTAATGCCTGCGAAG	qRT-PCR
*S* *l* *GA2ox2*	RT-SlGA2ox2-R	ACTTGATGGCTTCGGATTCGAG	qRT-PCR
*S* *l* *GA2ox4*	RT-SlGA2ox4-F	CTCATCGTTAATGCCTGCGAAG	qRT-PCR
*S* *l* *GA2ox4*	RT-SlGA2ox4-R	GATCAGCAGGCCCTGCCTTTAG	qRT-PCR
*S* *l* *GA2ox5*	RT-SlGA2ox5-F	GAACCTCATCGTTGAGGCCTGC	qRT-PCR
*S* *l* *GA2ox5*	RT-SlGA2ox5-R	GATGGCTTCGGATTCGAGTTTAC	qRT-PCR
*DELLA*	RT-DELLA-F	CGATGGTTACAGGGTGGAAGAA	qRT-PCR
*DELLA*	RT-DELLA-R	CAGGCGGAGGTAGCTATAAGTG	qRT-PCR

**Table A3:** Amino acid sequences of related GAMYB proteins in various species used in this study.

Protein	Species	Amino Acid Sequences
SlGAMYB1	*Solanum lycopersicum*	MSIKSETEERMTSKVDMDSPDEASGGDLGESVPLKKGPWTSAEDVILVDYVMTHGEGNWNAVQRHSGLARCGKSCRLRWANHLRPDLKKGAFTPEEEQRIVELHAKMGNKWARMAVELPGRTDNEIKNYWNTRIKRRQRAGLPIYPADISFMASQNKQNEELGAFSSADAQNPDVLGINNFEIPAVEFKKLELTHLLYPPQLADIPARSLLNDPVSNFLSQGHRAPYSSTYFLSTTYPAKRIRGSESVFSGSNGDLLNSLQYQNDGSLLAQAQAQPLDFSSYNHNLTYDDQRAISNIVPGGHAYLNGNSSSEPTWAMKLELPSLQNQTENWGSPHSALPSLDSVDILIQSPPAGHSESGSLSPSNSGLLDAVLHESQTMKASNDNSYQGNETSGNAVNNSCPDLKGCDIYGHPVSPLSQFSASVFSDYAPINESSLHEFPSMATMPGGEIKQEIGDLSPLDDEDNTSNQTIFSSPKTQHANNHLASKDPFGSCFFDDCDWDCKQIHAVTTSSGQANGHNSCSWDAISAMEATGRMRL
SlGAMYB2	*Solanum lycopersicum*	MSMTSESDDRMTSQDGVDSPSAEEACGGGNTGGGLPLKKGPWTSAEDAILVEYVTKHGEGNWNAVQKHSGLARCGKSCRLRWANHLRPDLKKGAFTPEEERHIIELHAKMGNKWARMAAELPGRTDNEIKNYWNTRIKRRQRAGLPIYPSDICFQSITENKQNEELGTFSSADSQYPDFFPMNYEIPAVEFKRLEFNQHLCPPALLDIPTGGILDIPGRSLLAQGLNSAYYSRSFLSTTP
AtMYB33	*Arabidopsis thaliana*	PAKRIRGSESLFSGLNGDCSPSKNDVSFSTCHQHQDDGSLLAQSMGFSSSFNQNLTSDYHPSSLGVIPGSHALLNGHTSSSEPSWAKKLELPSLQSTIASWGLVTSPLPSLDSVDTLIQSPPTEHTESCNLSPRNSGLLDAVLHESQTMKASKSILHQENSGDVVDNSCPDLHMTEWGQHGDPISPLGHSAASVFSEYTPTSGSSSEEPQLVTMPACKVKQEKFDYGPYDGKDDASNLICPRPDFLLESNCFGHMQNTVRSIWY
AtMYB65	*Arabidopsis thaliana*	MSYTSTDSDHNESPAADDNGSDCRSRWDGHALKKGPWSSAEDDILIDYVNKHGEGNWNAVQKHTSLFRCGKSCRLRWANHLRPNLKKGAFSQEEEQLIVELHAKMGNRWARMAAHLPGRTDNEIKNYWNTRIKRRQRAGLPLYPPEMHVEALEWSQEYAKSRVMGEDRRHQDFLQLGSCESNVFFDTLNFTDMVPGTFDLADMTAYKNMGNCASSPRYENFMTPTIPSSKRLWESELLYPGCSSTIKQEFSSPEQFRNTSPQTISKTCSFSVPCDVEHPLYGNRHSPVMIPDSHTPTDGIVPYSKPLYGAVKLELPSFQYSETTFDQWKKSSSPPHSDLLDPFDTYIQSPPPPTGGEESDLYSNFDTGLLDMLLLEAKIRNNSTKNNLYRSCASTIPSADLGQVTVSQTKSEEFDNSLKSFLVHSEMSTQNADETPPRQREKKRKPLLDITRPDVLLASSWLDHGLGIVKETGSMSDALAVLLGDDIGNDYMNMSVGASSGVGSCSWSNMPPVCQMTELP
OsGAMYB	*Oryza saliva*	MSYTTATADSDDGMHSSIHNESPAPDSISNGCRSRGKRSVLKKGPWTSTEDGILIDYVKKHGEGNWNAVQKHTSLARCGKSCRLRWANHLRPNLKKGAFSQEEEQLIVEMHAKMGNKWAQMAEHLPGRTDNEIKNYWNTRIKRRQRAGLPLYPPEIYVDDLHWSEEYTKSNIIRVDRRRRHQDFLQLGNSKDNVLFDDLNFAASLLPAASDLSDLVACNMLGTGASSSRYESYMPPILPSPKQIWESGSRFPMCSSNIKHEFQSPEHFQNTAVQKNPRSCSISPCDVDHHPYENQHSSHMMMVPDSHTVTYGMHPTSKPLFGAVKLELPSFQYSETSAFDQWKTTPSPPHSDLLDSVDAYIQSPPPSQVEESDCFSSCDTGLLDMLLHEAKIKTSAKHSLLMSSPQKSFSSTTCTTNVTQNVPRGSENLIKSGEYEDSQKYLGRSEITSPSQLSAGGFSSAFAGNVVKTEELDQVWEPKRVDITRPDVLLASSWLDQGCYGIVSDTSSMSDALALLGGDDIGNSYVTVGSSSGQAPRGVGSYGWTNMPPVWSL
CaGAMYB1	*Capsicum annuum*	MYRVKSSDCDMIHMDSVADDGSSGGSHRGGGKKGWTSADAIVDYVKKHGGNWNAVKNTGRCGKSCRRWANHRNKKGATARIIHSKMGNKWARMAAHGRTDNIKNYWNTRIKRCRAGIYTSVCNSSNDCSSDDCGNSNDNANGYDTCDNIANSAYAHSAVSISNGSASKSCSMDVNTGMKSDGVGSDTINGVISSVDSNDSKKAVGDYHANSTSKIIAGGANGSHANGNSASRTSGKMSDTSDNSWKYTVAATVDYSAATSVKSCASRNSGIHATRSGKNTSVISSSSSVGTCNTTVSDMCYWHGNDCASGNSTSTVSAASDISKVSASTSMGSGVMGKYGDTSHNRDASGNTADSVNNAIAMGNDSIDCRVGDGIMNSSSWSNMHACMSK
CaGAMYB2	*Capsicum annuum*	MSITSETDDMMTSKVDIDSPDEASGGESVPLKKGPWTTVEDAILVDYVMTNGEGNWNAVQRHSGLARCGKSCRLRWANHLRPDLKKGAFTPEEERRILELHAKMGNKWARMAAELPGRTDNEIKNYWNTRIKRRQRAGLPVYPPDISFLANQNKQNEELGAFSSVDAQNSNVLGINNFEIPAVEFKNLQLDHLLYPPPLGEIPAVSSFLAQGHRAPYGSTYLLSTMHPSKRIRGSESMFSGSNGDLLLSSSQYHNGGSLLAQPLGFSSYNHHLTYDDDRSFSSVVHGGHACLNGNSSSSEPTWAMKLELPSLQNQTANWGSPPSPLPSLESDDILIQSPPAGNSESGSLSPSNSGLLDAVLYESQTMKASNDNSHQGKETSGDAVNKGWESYGDPVSPLHHFSASVFGEYTPVNGSSLHEFPSVATMPGCKIKKEIGDLAPLDENDDSLNQTIFSSPKTQHAKNSLALKEVISSGFFDDCGWDCKQIRAVATSSGQACGRSSWDAMSAV
CsGAMYB1	*Cucumis sativus*	MSMTSESDDRMASQDGVDSPSVEEACGGGITGGGLPLKKGPWTSAEDAILMDYVTKHGEGNWNAVQKHSGLARCGKSCRLRWANHLRPDLKKGAFTPEEERRIIELHAKMGNKWARMAAELPGRTDNEIKNYWNTRIKRRQRAGLPIYPPDISFQAISENKQNEELGAFSSTDSQYPDFLPMNNFEIPAVEFKRLEFNKQLCPPALLDIPNGGILDIPGRSLLAQGLNSAYYSRSFLSTMPPAKRIRGAESLFSGLNGDCSPSKNEGSFPTCHQYQDDGSLLAQSMGFSSSFNQNLTSVHHPSSSGVIPGSHAPLNGKTSSSEPLWAEKLELPSFQSQMASWGLSSSPLPSLESVDTVIQSPPTEHTESCNLSPRNSGLLDAVLYESQTMRASKSILHQENSGDVVDNSCPDLHETGWETYGDPISPLGHSAASVFSEYTPTSGSSPEEPQLVTMPGCKVKQEKFDFGPYDGKEDASDLIFSRPDYLLESNCFGHMQKTARSIWH
HvGAMYB	*Hordeum vulgare*	MRHPKNEIEDNLPSQDQTLSPLLDEDSGGNASGIILKKGPWTSAEDEILIEYVKKHGEGNWNAVQKHSGLSRCGKSCRLRWANHLRPNLKKGAFTAEEEHLIIELHAKMGNKWARMAGHLPGRTDNEIKNYWNTRIKRRQRAGLPLYPPEVCLRTWQALQQTQDSGGSTVVDTDHHDLLRSNSYDIPDVTFHSLKPQSALSYMPELPDISSCMLKRGLDTSQYCNLVQPTFHRQKRFRDSASLFPGPDGSVKTPFHQFEDNSYSQAAQSFGTPFAHESNPTTKNAMSFGSFEGSHSLTNGNSSASQHSKETEKLELPSLQYPETDLTSWDTTIQPAMFESVDPFIQSTPTFVLAPDRTSPCHSGLLESLVYSKTMGPKNHPSDKNSNSCSVTPGDVTDSYNMAASKTEIDDYTEVISPFGHSTSSLFSECTPISATGSSYEDPTLTEAFSGSHVKSEPFDHAWTPDREKAAKSRVNFARPDALLASDWHDRSSGIVEDTTNVTDAISLLLGDDLAADYEHFPNGISTTHSAWGLDSCSWNNMPAVCHMSDLP
